# Cicatrical Contraction from Burns, Involving the Face

**Published:** 1874-12

**Authors:** Gurdon Buck


### ARTICLE VI.

### *Cicatricial Contractions from Burns, Involving the Face*

BY GURDON BUCK, M. D.

H. C. W., æt. four years and seven months, resident of New Jersey, of sound constitution and enjoying good health, was extensively burned when sixteen months old by over turning upon himself a kerosene study lamp. At the expira tion of about fourteen months the burnt surfaces had mostly healed. The parts involved in the cicatricial contractions which resulted from the burns were the left half of the face and scalp, and the dorsal surface of the left hand and forearm. When I first examined him, in February, 1872, his condition was as follows .—The left half of the scalp, as far back as the occipital region, was bare, and the surface presented a pale, shining, cicatricial aspect; the skin, however, was pliable, and moved freely on the underlying parts. The left ear, though diminished in size by the loss of its rim, retained its natural shape, and was not adherent to the scalp. The skin covering the left half of the forehead and temple, though cicatricial on its surface, was still movable on the subjacent parts. The outer half of the left upper eyelid was everted to an extreme degree, and spread out upon the eyebrow; the inner half of its tarsal border alone came into contact with the lower lid. The conjunction covering the everted portion of the lid being relaxed and swollen, filled up the space between the lids when closed, and thus protected cornea, which otherwise would have become opaque from constant exposure. The eye-lashes, as well as the tarsal edges of both lids, had escaped injury. The eyebrow was denuded of hair. The eyeball itself had sustained no damage, the cornea retaining its natural lustre, and vision being unimpaired. The surface of the left cheek and side of the nose was cicatricial, and the contraction consequent thereupon had drawn the underlid away from contact with the eyeball, but without producing any eversion of its tarsal margin. The left angle of the mouth was somewhat drawn upward by a vertical fold of cicatricial skin upon the cheek immediately above it. In consequence of the condition of the lids of the left eye, patient habitually held his head inclined forward and to the left side, so as to avoid direct exposure to bright light, and had thereby acquired a peculiar expression of countenance. A description of the left hand and forearm will be reserved to a subsequent part of the narrative.

On the 6th of February, 1872, an operation was performed in the presence of Dr. James A. Davis, the family physician, from Bloomfield, New Jersey, Drs. John Beekman and Thomas Satterthwaite, and Prof. A. C. Post, M. D.

*First Operation.*—The object of this operation was to restore the upper lid of the left eye to its normal relations and functions. It was attempted as follows:—Two incisions were started from a single point high up on the forehead, above the middle of the left eyebrow, and continued downward in lines diverging from each other and terminating, one at either canthus of the eye. The inverted V-shaped patch of skin, included between these incisions, was dissected up from the pericranium as low down as the bony margin of the orbit. The upper lid was thus liberated and brought down so as to allow its tarsal margin to be adjusted in contact with that of the underlid. Before proceeding further, a transverse fold of the redundant conjunctiva, lining the upper lid, was excised as far back from its tarsal margin as possible. With the descent of the upper lid, the inverted V-shaped patch of skin, which had been raised from the forehead, was brought down and secured at a lower level by a pin suture inserted on either side, together with additional thread sutures above the pin sutures. The surface left bare on the forehead by the descent of the patch was closed by approximating the opposite edges of the wound, and securing them in contact by thread sutures. To relieve these last sutures from tension, parallel incisions were made through the skin on either side, the one over the left temple being three inches, while the other, toward the middle of the forehead, was only one inch in length. The edges of these incisions yawned wide apart, and the desired relief was thus obtained. The bare surfaces left were coated with a collodion crust, formed by applying, first, a layer of dry lint, and then over it a second layer of lint saturated with collodion, which soon stiffens and adheres closely to the surrounding surface. In order to hold the tarsal edges of the lids more exactly in coaptation, a beaded silver-wire clump suture was passed in a vertical direction through both lids towards the outer canthus, and out of the way of the cornea, and left in situ for three days. Wet dressings were avoided, and a layer of woven lint of double thickness was kept applied to the parts. Moderate febrile reaction followed the operation, but subsided on the third day. On the fourth day the last sutures were removed. Sloughing, however, had already taken place, and had involved about three-fifths of the transplanted patch. Healthy suppuration succeeded the separation of the sloughs, and the ulcerated surface progressively diminished in size. The collodion crust separated from the forehead on the sixth day, and from the left temple on the tenth, leaving healthy granulating surfaces to heal by cicitrization. The eyeball had in no way suffered from the presence of the silver wire which traversed the lids. Patient rapidly regained his spirits, and at the end of one week resumed his accustomed amusements, enjoyed his meals, and rested well at night. The sloughing which had taken place was no doubt to be attributed to the cicatricial condition of the transplanted patch of skin. The ulcerated surface above the left eyebrow progressively diminished in size, and at the end of the fourth week measured one inch in its transverse diameter, by three-fourths of an inch vertically. If left to itself it was feared that the contraction consequent upon cicatrization might produce, to a greater or less degree, the original eversion of the upper lid. To prevent this it was determined to transfer a portion of sound skin from the right half of the forehead and engraft it upon the ulcerated surface. The operation for accomplishing this object was performed on the 8th of March.

*Second Operation.*—The ulcerated spot itself was prepared by first excising the granulating surface down to the level of the pericranium, with scissors, applied flatwise, and then paring afresh the edges, and everting them slightly. A transverse incision was then carried across the forehead, on a line continuous with the lower margin of the spot just prepared, and as far as the inner extremity of the right eyebrow. The upper edge of this incision was dissected up to afford a bare surface of one finger's breadth, which would be continuous with and form a part of the space above the eyebrow. A pattern, cut from oiled silk, of the size and shape of the space just prepared, was applied upon the right of the forehead, in a vertical position, with its base resting upon the inner half of the eyebrow, and its free extremity involving the hairy scalp above, which had been previously shaved clean. An incision was then carried around the margin of the pattern, and the included underlying patch of skin was dissected up from the pericranium, but left connected, for support, at the margin of the orbit. The pedicle of the patch, at its inner edge, toward the median line, was adjacent to the bare surface which it was intended to cover. Additional room had to be made for the transfer of the patch, by dissecting up the skin from the forehead, above the nose, and displacing it toward the left side, where it remained attached, and was reserved for subsequent use. The patch was now brought down edgewise from right to left, and adjusted accurately to the edges of the space prepared for it, with sutures inserted close together. In order to utilize the portion of skin displaced from above the nose, for the purpose of covering the surface left bare on the right half of the forehead, it was carried upward from left to right and adjusted to the lower part of the bare surface by means of sutures. The remaining upper portion of the bare surface was coated over with a collodion crust, and left to heal by granulation. Before proceeding to the operation just described, a transverse fold was excised, for the second time, from the still redundant conjunctival lining of the upper lid, and the lids themselves were secured together by a single thread suture inserted through the skin alone, near their tarsal edges, and toward the outer canthus. A strip of woven lint, saturated with collodion, was applied across the outer half of the closed lids, to afford additional support. The entire operation occupied one hour and a half, and was well borne, the loss of blood having been inconsiderable. A layer of woven lint, of double thickness, was spread with cerate, to prevent its sticking to the surface, and laid upon the forehead. Elixir opii, gutt. x., and weak brandy and water were directed. March 10.—*Second day.*—Slight febrile reaction and moderate inflammatory tumefaction. Removed three pins and changed the yarn on the remaining ones. 12th.—*Fourth day.*—All the sutures have been removed in succession, including the one holding the lids in contact. To supply the place of this last suture strips of adhesive plaster were applied. Primary union has taken place at almost all points, and with any sloughing. 20th. —*Twelfth day.*—The collodion crust came off from the forehead, exposing healthy granulations at the circumference of the sore, but in the centre a brownish patch of sloughing pericranium, which had been unintentionally divided in the operation. No exfoliation of bone, however, followed the sloughing of the pericranium ; healthy granulations covered the spot, and cicatrization followed. Iron and quinine were ordered as a tonic.

*Third Operation.*—The patient being in excellent condition, from a stay of three weeks in the country, a third operation was performed on the 20th April, for the purpose of removing a conspicuous distortion of the left angle of the mouth. An incision, commencing at a point on the left cheek, bordering on the middle of the nose, was carried downward and outward across the cheek to a point a little anterior to the angle of the jaw. In its course the incision divided the cicatricial fold which distorted the angle of the mouth, and so allowed the latter to regain its natural shape. The edges of the incision, after having been dissected up, receded from each other and left a space between them of about one finger's breadth. To fill this up with sound skin, the following method was adopted : A patch of skin of the required shape and size was dissected up from the side of the neck below the edge of the jaw, its free extremity being below the symphysis, and its pedicile of attachment adjoining the space to be filled up. This patch was then transferred edgewise to its new locality, and then accurately adjusted by sutures. The wound left on the neck was closed by approximating its opposite edges, and securing them together with sutures. The treatment was the same as after the previous operations. Union failed to take place, and sloughing of about three-fifths of the patch followed.

On the fourth day the slough separated, and healthy suppuration succeeded. It was now important to prevent shrinking of what remained of the patch, and to maintain it in place. This was done by adhesive straps carefully adapted and frequently removed. April 25—A mild attack of erysipelas developed itself upon the left ear and neighboring surface of the scalp, but soon passed off without any serious consequences. From this time his general health improved, and cicatrization of all the sore surfaces progressed steadily till June 8th, when all had finally healed. The result of the last operation, notwithstanding the loss of so large a portion of the transplanted patch of skin, was not without some improvement of the angle of the mouth. As no further operations would be undertaken till the autumn, patient's nurse was instructed to manipulate the parts upon the forehead and left cheek daily, so as to increase their pliability and prevent contraction from taking place. The good effect of these manipulations was manifest when patient returned in October to spend the winter in the city with his family.—*Medical Record.*

---

